# Nanoarchitectonics meets cell surface engineering: shape recognition of human cells by halloysite-doped silica cell imprints

**DOI:** 10.3762/bjnano.10.176

**Published:** 2019-09-04

**Authors:** Elvira Rozhina, Ilnur Ishmukhametov, Svetlana Batasheva, Farida Akhatova, Rawil Fakhrullin

**Affiliations:** 1 Institute of Fundamental Medicine and Biology, Kazan Federal University, Kreml uramı 18, Kazan 420008, Republic of Tatarstan, Russian Federation

**Keywords:** cell surface engineering, cell-recognising imprints, halloysite nanotubes, nanoarchtectonics

## Abstract

Cell surface engineering, as a practical manifestation of nanoarchitectonics, is a powerful tool to modify and enhance properties of live cells. In turn, cells may serve as sacrificial templates to fabricate cell-mimicking materials. Herein we report a facile method to produce cell-recognising silica imprints capable of the selective detection of human cells. We used HeLa cells to template silica inorganic shells doped with halloysite clay nanotubes. The shells were destroyed by sonication resulting in the formation of polydisperse hybrid imprints that were used to recognise HeLa cells in liquid media supplemented with yeast. We believe that methodology reported here will find applications in biomedical and clinical research.

## Introduction

Nanoarchitectonics has recently emerged as a “post-nanotechnology era” paradigm in the directed fabrication of functional materials [1]. It widely employs atom and molecule manipulation and self-organisation of nanoscale particles [2]. Engineering of cell surfaces with various nanoscale materials has been recognised as a powerful means to attenuate the intrinsic properties of microbial and eukaryotic cells [3]. In particular, nanostructured composite shells (both hard and soft) deposited onto live cells have been shown to render the cells with novel mechanic and chemical functionalities [4–6]. In line with the concepts of nanoarchitectonics, cell surface engineering relies on the self-assembly of miniature building blocks to form biomimetic soft or rigid shells to encapsulate live cells rendering them with additional functionalities [7]. In general, there are three principal routes to engineer the cell walls or membranes of live cells: 1) deposition of charged or neutral polymers (that can be doped with nanoscale inorganic particles) [8–9]; 2) direct anchoring of inorganic nanoparticles to cell surfaces [10–11]; 3) fabrication of “hard” inorganic shells mimicking natural eggshells [12]. Synthetic polymers can be grafted onto the surface of individual cells using atom-transfer radical polymerization [13]. The versatility of cell surface engineering methods has led to intersections between these routes yielding functionalised cells with multiple functionalities [14]. Relatively solid microbial cells with cell walls as well as soft mammal cells (including human cells) were used in cell surface engineering [15]. Surface-engineered cells have found applications in whole-cell biocatalysis [16], cell therapy [17], magnetic cell delivery [18], fabrication of multicellular assemblies [19], cell protection [20–21], biosensors [22] and tissue engineering [23]. Shells derived from cells templates offer other fascinating opportunities due to their cell-mimicking geometries, for example, a novel class of bioinspired colloid particles was fabricated recently. Colloid antibodies were produced via the formation of solid silica shells doped with gold nanoparticles on bacterial cells. The cells were chemically decomposed, while the empty shells were broken by ultrasound and later used for shape-based recognition and killing of bacteria [24–25].

Inspired by the previous reports on the fabrication of colloidal cell imprints capable of microbial cell recognition [24–25], we have developed a nanoarchitectonics-based technology to produce imprints recognising human cells. To do so, we resorted on forming silica-based solid shells and reinforcing these shells with halloysite nanotubes. Halloysite, a naturally occurring biocompatible clay, is a promising candidate for the fabrication of various functional composite materials [26]. The anisotropic shape (hollow tubules having lengths from 300 nm to 1–2 µm, 50–70 nm diameter, and 20 nm lumen) and surface chemistry (outer surface of SiO_2_, inner surface of Al_2_O_3_) make these nanotubes ideal carriers for novel catalysts, polymer fillers, drug-delivery vehicles and tissue engineering scaffolds [27]. Halloysite nanotubes derived from various geological deposits differ in their mesoscopic structures [28], allowing to choose the clay nanotubes most suitable for a desired application. The positively charged nanotube lumen can be loaded with anionic molecules (including bioactive compounds), and the loading efficiency can be significantly increased by using vacuum pumping [29].

Halloysite has already shown its potential in cells surface engineering of microbial cells [30–31]. Here we used halloysite as a dopant for artificial silica shells deposited on viable human HeLa cells. Halloysite nanotubes were chosen as dopant because of their biocompatibilty and rather large lumen sizes suitable for loading various drugs and even enzymes [32]. In the future, the procedure developed here can be extended to other nanotubular particles such as boron nitride or imogolite nanotubes, which are also considered as safe materials for living organisms [33]. Recently, water-dispersed thermo-responsive boron nitride nanotubes were obtained by their functionalisation with poly(*N*-isopropylacrylamide), which can widen their biomedical applications [34]. After fabrication, the cells were bleached to produce hollow cell-shaped imprints. These imprints, in turn, were utilised to recognise HeLa cells in suspension. Importantly, the silica/halloysite imprints based on human cells were selective and did not interact with microbial cells of comparable sizes such as yeast cells.

## Results and Discussion

**Figure 1:**
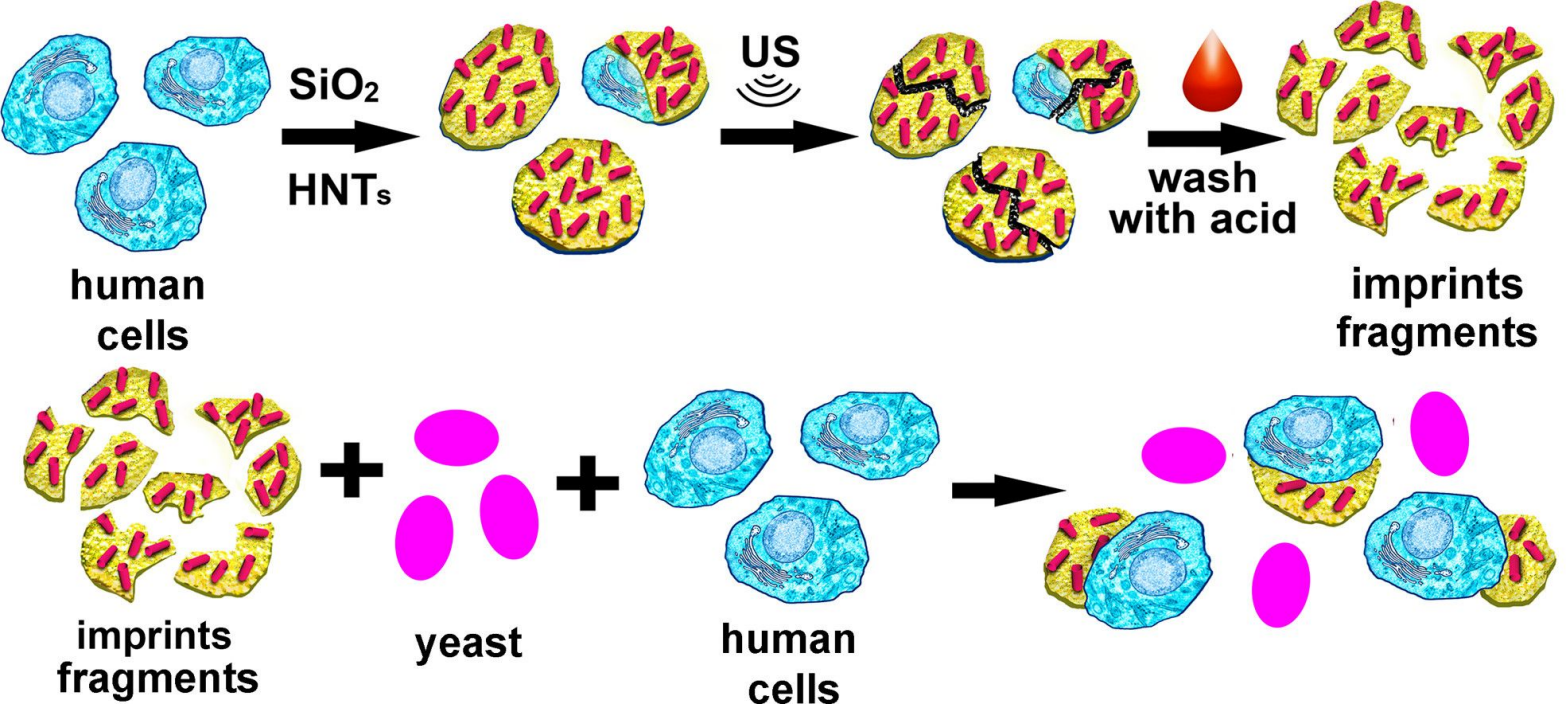
Production of silica/halloysite cell-mimicking imprints and recognition of human cells by the imprints in HeLa/yeast cells mixture. The imprints were obtained by destruction of inorganic shells deposited on live cells.

Our experimental strategy is schematically depicted in Figure 1. Following the nanoarchitectonics paradigm [35], we produced composite inorganic shells around human HeLa cells, using a polyelectrolyte nanolayer as a means to facilitate the formation of silica/halloysite cell-mimicking imprints. Then the shells were disintegrated using sonication, while the cell debris was dissolved by acid washing to produce polydisperse silica/halloysite cells imprints. Next, these imprints were utilised to selectively recognise HeLa cells in cell growth media supplemented with yeast cells.

**Figure 2:**
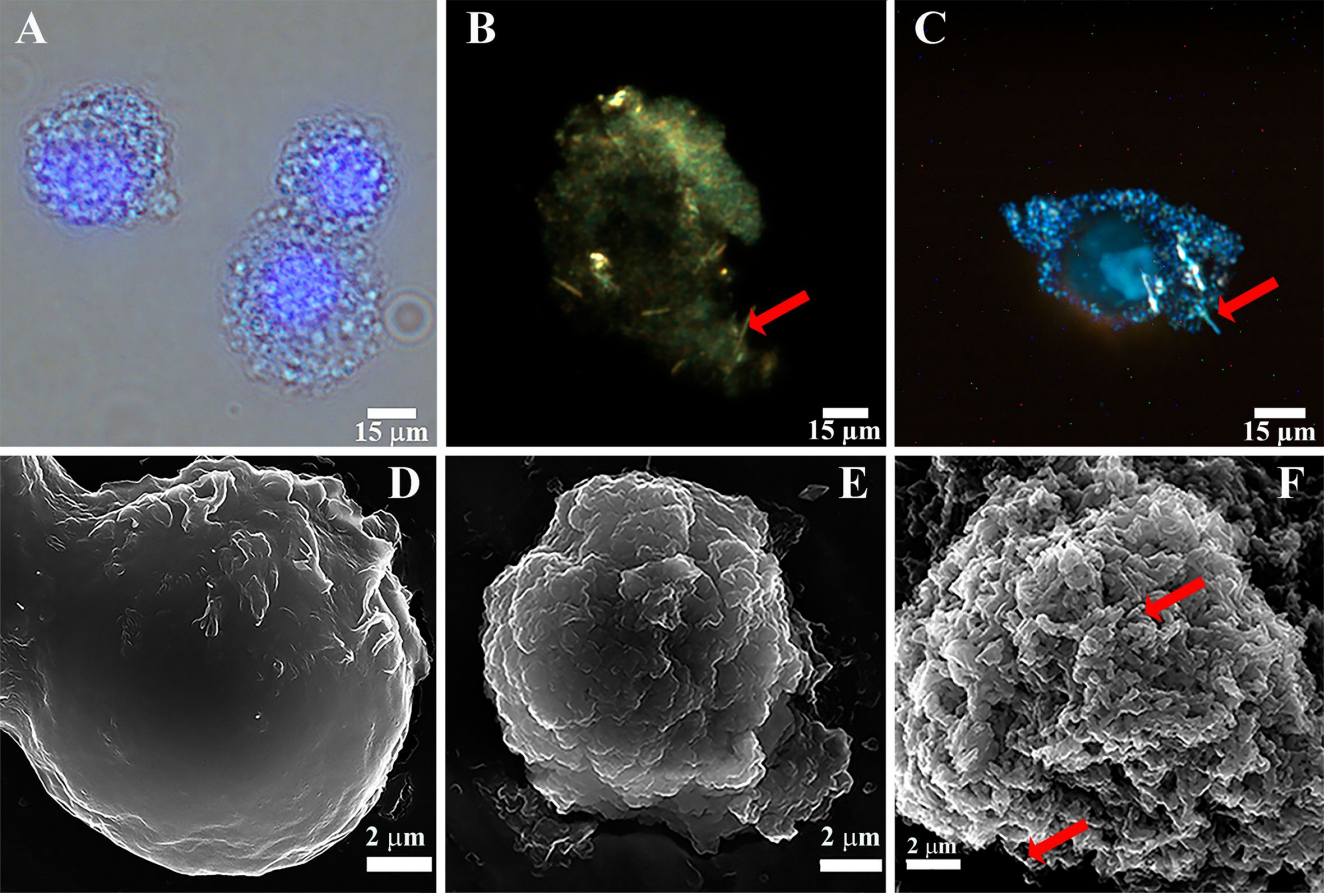
(A) optical/fluorescence microscopy image of live HeLa cells coated with halloysite-doped silica shells (nuclei are stained with DAPI); (B, C) dark-field microscopy images of live HeLa cells coated with halloysite-doped silica shells; scanning electron microscopy images of (D) intact HeLa cells, (E) HeLa cells coated with pure silica shells and (F) HeLa cells coated with halloysite-doped silica shells. Red arrows indicate halloysite nanotubes.

HeLa cells (having originally a negative zeta potential of ca. −10 mV) were first coated with a single layer of poly(acrylamide-*co*-diallyldimethylammonium chloride (P(AAm-*co*-DADMAC)) to reverse the surface charge of HeLa cells (the zeta potential after P(AAm-*co*-DADMAC deposition was ca. 46 mV). Positively charged cells were then subjected to a mixture of silicic acid derivatives (produced by mixing tetraethyl orthosilicate with HCl) and halloysite nanotubes (2.5 mg·mL^−1^) for 10 min. In several experiments, halloysite-free silica shells were obtained following a previously published protocol [24–25]. Silica/halloysite-decorated HeLa cells were then imaged in situ with optical fluorescence microscopy. A typical image is shown in Figure 2A demonstrating the preserved cell morphology and characteristic nuclear DAPI stain. Next, we imaged the silica/halloysite-decorated HeLa cells with dark-field microscopy (Figure 2B,C) to confirm formation and integrity of the inorganic layer. Dark-field microscopy at 1000× magnification is expected to resolve the rod-like shapes of halloysite [36]. This was confirmed in this study demonstrating the elongated tubular structures within the silica shells deposited around perinuclear areas, as shown in Figure 2C.

**Figure 3:**
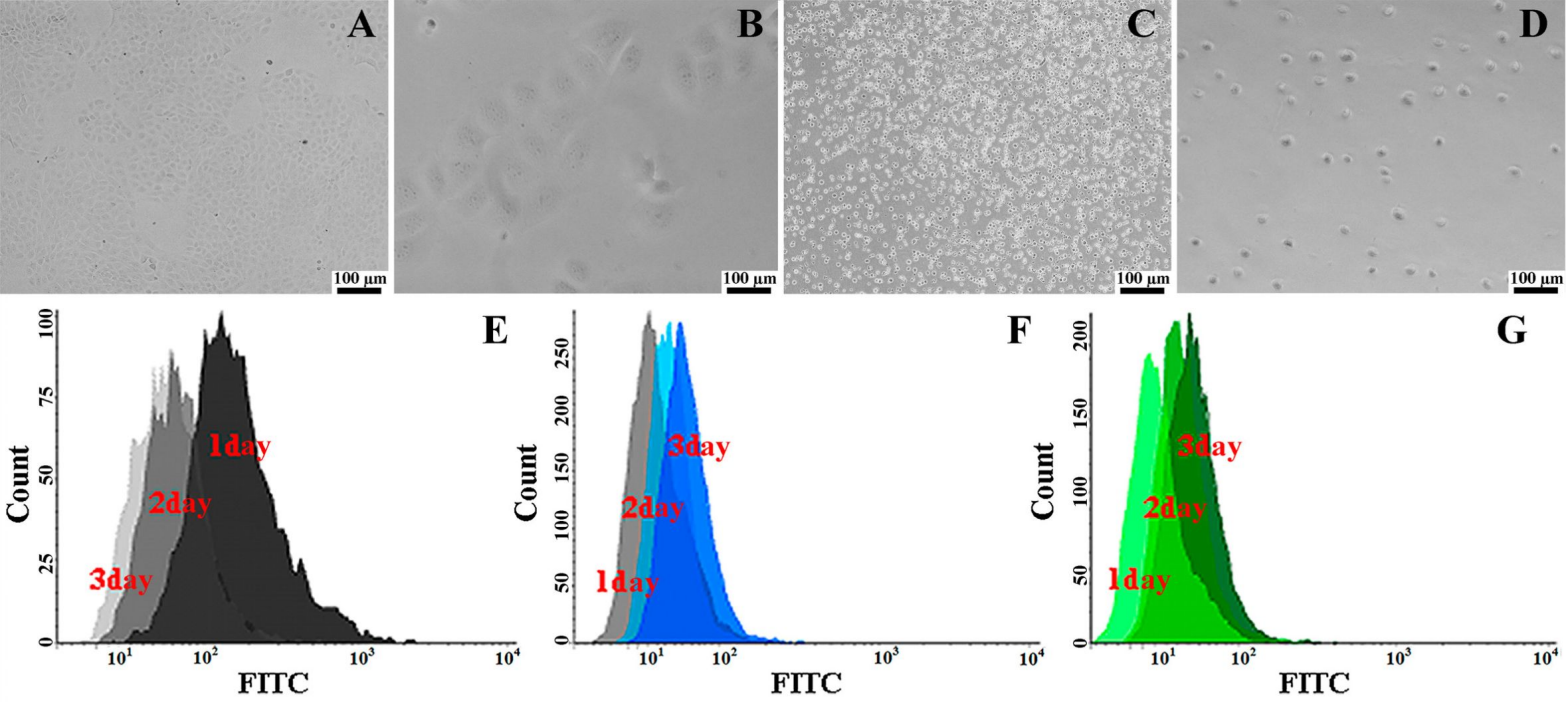
Optical microscopy images demonstrating the cultivation for 24 h of (A, B) substrate-attached intact HeLa cells and (C, D) HeLa cells coated with halloysite-doped silica shells; flow cytometry monitoring of cell division for three days of CFSE-stained (E) intact HeLa cells, (F) silica-coated HeLa cells and (G) HeLa cells coated with halloysite-doped silica shells.

Scanning electron microscopy (SEM) was employed to investigate the morphology of the halloysite-doped and halloysite-free silica shells deposited onto HeLa cells. Suspended cells were deposited onto glass substrates, fixed with formaldehyde, sputter-coated with a thin gold layer and then imaged using a Hitachi SU8000 microscope. As shown in Figure 2D–F, the typical smooth surface topography of HeLa cells was changed drastically by the deposition of either pure or halloysite-doped silica. Analysing the cell diameter in the SEM images of intact and silica/halloysite-coated cells, we estimated the thickness of the inorganic shells (ca. 2 µm). In general, the shells were quite resilient and not prone to any mechanical damage unless subjected to a significant impact. This was indirectly confirmed in viability evaluation experiments. First, we tried to cultivate the cells in a regular way (24 h) by seeding them onto cell culture plates. Uncovered HeLa cells, as expected, were able to adhere and subsequently colonise the substrates (Figure 3A,B), whereas the cells decorated with halloysite-doped silica shells did not adhere or proliferate, which was confirmed by optical microscopy (Figure 3C,D). One might expect that the deposition of silica/halloysite shells kills the HeLa cells, which lack any rigid cell protective structure such as cell walls in microbial cells [37], and that therefore no visual cell growth occurs. However, flow cytometry-based cell proliferation monitoring performed with cells stained with 5(6)-carboxyfluorescein diacetate *N*-succinimidyl ester (CFSE) dye has confirmed that cells coated with pure silica or silica/halloysite display a similar cell proliferation pattern as intact HeLa cells (Figure 3F,G), confirming the viability of the encapsulated cells. CFSE-labelled HeLa cells divide, thus the overall fluorescence intensity decreases. In case of silica-encapsulated HeLa cells there was a prominent decrease in fluorescence intensity during day 1 of the observation, which we attribute to the inhibition of proliferation by the silica shells. On day 3, however, the fluorescence intensity in silica-coated cells was even higher than in the control cells, apparently due to the partial destruction of the shells and the release of HeLa cells.

**Figure 4:**
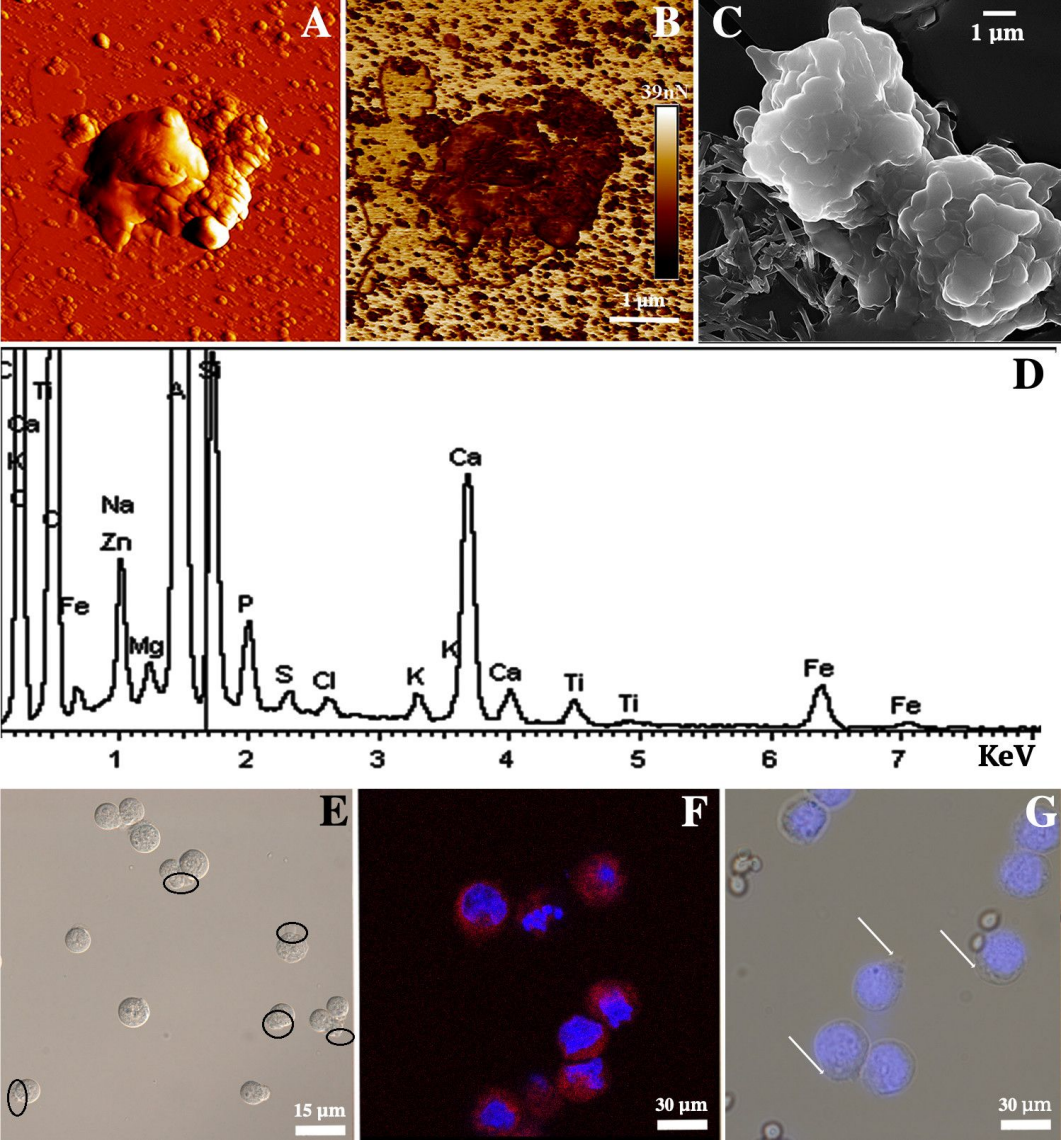
Atomic force microscopy (PeakForce Tapping mode) images of inorganic silica/halloysite imprints templated on HeLa cells: (A) topography image, (B) non-specific adhesion map; (C) scanning electron microscopy image of inorganic silica/halloysite imprints templated on HeLa cells; (D) EDX spectrum taken from the sample shown in (C), demonstrating the typical silica and halloysite elemental distribution; (E) optical and (F) confocal microscopy images demonstrating the recognition of HeLa cells with cell-templated imprints (cell nuclei stained with DAPI, imprints with rhodamine B in panel (F); (G) optical microscopy image of selective recognition of HeLa cells by the imprint in a mixture of human cells with yeast cells.

Although non-compromised viability is an important feature in any cell surface engineering investigation, in this study we were more concerned with the fabrication of imprints that are able to recognise human cells. To do so, we destroyed the cell-in-shell structures obtained by drying, resuspending in water and sonicating for 10 min. Then the organic cell debris was removed by treatment with aqueous HNO_3_ and HCl mixture (3:1) and washed thoroughly with water. This procedure yielded polydisperse (100 nm to 1 µm) cell-templated imprints with diverse morphology. Typical AFM and SEM images of the imprints are shown in Figure 4A–C. We have also used EDX spectroscopy to investigate the elemental composition of the silica/halloysite imprints (Figure 4D), confirming the typical elemental composition characteristic for halloysite. A strong peak of Al in the EDX image supports the presence of halloysite in the imprint, because Al is a major constituent of halloysite nanotubes.

The main goal of this study was to demonstrate the recognition of human cells by cell-templated imprints in a similar way as was reported previously for bacteria recognition [24–25]. To do so, we have mixed the imprints (0.1 g·mL^−1^) with suspended HeLa cells (10^6^ mL^−1^) in growth media (Figure 4E). After 30 min of incubation, we found that more than 60% of the HeLa cells were recognised by the imprints. Additionally, for a better visualisation of the cell recognition events the imprints were labelled with rhodamine B and then added to DAPI-stained HeLa cells (Figure 4F). It is likely that the recognition event is facilitated by the nanostructured internal surface of the imprint built from halloysite nanotubes, which may increase the effective contact area between the cell surface and the imprint surfaces. In previous reports [24–25], the recognition event was apparently facilitated by the shape matching between the bacteria with rigid cell walls and the relatively uniform hollow imprints. In our study large-scale shape recognition is not likely to occur because HeLa cells are labile and do not keep the shape as well as bacteria do. We therefore believe that the recognition is based on a small-scale interaction between the inner surface of the imprints retaining the local cellular membrane shapes and cells. In this case, halloysite nanotubes may have a significant effect on the reproduction of the local cell non-uniformities helping the subsequent recognition to happen. Finally, to demonstrate the selective recognition, we prepared mixtures of HeLa cells with yeast cells and then added HeLa cell-templated imprints. HeLa cells were pre-stained with a DAPI nuclear dye for better visualisation of mammal cells in a mixture with fungi cells. As shown in Figure 4G, only HeLa cells were recognised by the imprints, while yeast cells were ignored. Although this does not guarantee an equally effective recognition of other mammalian cells (i.e., in a mixture with HeLa cells), our results open new avenues for the fabrication of colloid particles capable of shape-based recognition of human cells. Viral and chemical transformation of cells induce changes in the architecture of the membrane [38], and tumor cells have many more microvilli than non-dividing normal cells [39]. These differences in the surfaces characteristics of cancer and normal cells can potentially be used for designing new methods for the selective recognition of normal and tumor cells. Future research is needed to thoroughly investigate the capabilities of our method to template imprints and then recognise the target cells depending on species or cell type.

## Experimental

### Materials and reagents

Tetraethoxysilane (TEOS), poly(acrylamide-*co*-diallyldimethylammonium chloride) (P(AAm-co-DADMAC), rhodamine B, poly(allylamine hydrochloride) (РАН) were purchased from Sigma-Aldrich. Millipore water (specific resistivity 18 MΩ·cm at 25 °C) was used in all experiments. Halloysite nanotubes (HNTs) of 95–98% purity were obtained from Applied Minerals Inc.

### Cell culture

The human cervical carcinoma (HeLa) CCL-2 cell line was obtained from the American Type Culture Collection (ATCC, USA).The cells were cultured under standard culture conditions (5% CO_2_ at 37 °C) in Dulbecco’s modified Eagle’s medium (DMEM) containing 10% heat-inactivated foetal bovine serum (FBS, Invitrogen, USA).

### Fabrication of imprints using HeLa cell templates

The silica shells on individual HeLa cells were fabricated by biosilification [3]. To pre-functionalise the cells with P(AAm-*co*-DADMAC), the cells were trypsinised, collected, re-suspended in the medium to 10^6^ cells·mL^−1^and mixed with PAAm-*co*-DADMAC (1 wt %). After 10 min of incubation with the polycation solution, the cells were washed thrice with buffer to remove excess polyelectrolyte. Next, a thin silicon film was formed on the pre-functionalised cells. The working solution was prepared by mixing 1 part of TEOS with 0.1 parts of deionized H_2_O and 0.01 parts of 1mM HCl for 20 min at room temperature, similarly to the approach described elsewhere [40]. The halloysite nanotubes were added to silicic acid derivatives to a final concentration of 2.5 mg·mL^−1^. Then, the acid derivatives were mixed with the cells in serum-free medium (1:50 v/v) for 10 min on a rotator. The cells@SiO_2_-HNTs were washed five times with Milli-Q water, and the sediment was dried for 12 h at 105 °C. Dried cells@-SiO_2_-HNTs were re-suspended in Milli-Q water and crushed using an ultrasonic bath for 6–8 min. To remove the cell debris from the silica-halloysite imprints the cells@-SiO_2_-HNTs fragments were centrifuged at 4500 rpm, the supernatant was removed, and 10 mL of a HNO_3_ and HCl mixture (3:1) was added to the precipitate. After 30 min the silica-halloysite imprints were separated, washed three times with Milli-Q, and studied with AFM and SEM.

### Cells recognition by imprints

The recognition of HeLa cells with imprints was visualised using bright-field optical microscopy (Axio Imager Z2, Carl Zeiss), and laser confocal microscopy (LSM 780: 405 nm and 633 nm lasers). For fluorescence microscopy imaging, inorganic cell imprints were incubated for 5 minutes in a solution of rhodamine B and then washed with distilled water until the water remained clear. After that, the inorganic imprints obtained from one million cells were placed in 1 mL of DMEM medium containing one million cells and incubated for 20–30 minutes. The nuclei of the cells were stained with 4′,6-diamidino-2-phenylindole (DAPI) according to the standard protocol. In order to check the specificity of the imprint binding to mammalian cells the imprints were placed in DMEM medium, containing one million Hela and one million yeast cells.

### Characterisation

Scanning electron microscopy (SEM) imaging of samples sputter-coated with gold was performed with a Hitachi SU8000 microscope equipped with energy-dispersive X-ray (EDX) spectrometer. The interaction of cells with inorganic shapes was also recorded with an atomic-force microscope (Dimension Icon, Bruker, USA) operating in a PeakForce Tapping mode in air. The cells incubated with imprints for 15 minutes were washed with buffer; the precipitate was kept in glutaraldehyde (Sigma) for 1 hour, then washed with buffer and Milli-Q. Standard silicon nitride ScanAsyst-Air probes (Bruker) with resonance frequencies in the range of 70 to 95 kHz and spring constant in the range of 0.4 to 0.8 N m^−1^ (nominal length 115 µm, tip radius 2 nm) were used. The images were collected in air at 0.8–0.9 Hz scan rate and 512–1024 lines per scan. Topographic and nanomechanical characteristics were obtained. The data was processed using Nanoscope Analysis v.1.7. software (Bruker). For dark-field microscopy imaging HeLa cells were fixed and the nuclei were stained with DAPI. The Cytoviva^®^ high annular aperture dark-field condenser attached to an Olympus BX51 upright microscope was used. The Olympus BX51 microscope was equipped with a fluorite 100× objective and Dage xL (Dage-MTI) CCD camera. Images were obtained using Exponent 7 software (Dage-MTI). The dark-field images were overlapped with transmission fluorescence images using the image processing software GIMP. Cell proliferative activity was examined by flow cytometry on the 1st, 2nd and 3rd day of cell co-incubation with imprints. The cells were stained with 5(6)-carboxyfluorescein diacetate *N*-succinimidyl ester (CFSE) (Invitrogen) as specified by the manufacturer and analysed using a BD FACS (USA) instrument.

## References

[1] Ariga K, Ahn E, Park M, Kim B-S (2019). Chem – Asian J.

[2] Ariga K, Ji Q, Nakanishi W, Hill J P, Aono M (2015). Mater Horiz.

[3] Park J H, Hong D, Lee J, Choi I S (2016). Acc Chem Res.

[4] Shi P, Zhao N, Coyne J, Wang Y (2019). Nat Commun.

[5] Chen Z, Ji H, Zhao C, Ju E, Ren J, Qu X (2015). Angew Chem, Int Ed.

[6] Guryanov I, Naumenko E, Konnova S, Lagarkova M, Kiselev S, Fakhrullin R (2019). Nanomedicine: NBM.

[7] Hong D, Lee H, Ko E H, Lee J, Cho H, Park M, Yang S H, Choi I S (2015). Chem Sci.

[8] Jonas A M, Glinel K, Behrens A, Anselmo A C, Langer R S, Jaklenec A (2018). ACS Appl Mater Interfaces.

[9] Emanet M, Fakhrullin R, Çulha M (2016). ChemNanoMat.

[10] Maheshwari V, Fomenko D E, Singh G, Saraf R F (2010). Langmuir.

[11] Rozhina E, Batasheva S, Gomzikova M, Naumenko E, Fakhrullin R (2019). Colloids Surf, A.

[12] Fakhrullin R F, Minullina R T (2009). Langmuir.

[13] Kim J Y, Lee B S, Choi J, Kim B J, Choi J Y, Kang S M, Yang S H, Choi I S (2016). Angew Chem, Int Ed.

[14] Naumenko E A, Dzamukova M R, Fakhrullina G I, Akhatova F S, Fakhrullin R F (2014). Curr Opin Pharmacol.

[15] Kim B J, Cho H, Park J H, Mano J F, Choi I S (2018). Adv Mater (Weinheim, Ger).

[16] Zhang Y, Zhang S, Sun L, Yang Q, Han J, Wei Q, Xie G, Chen S, Gao S (2017). Chem Commun.

[17] Choi D, Lee H, Kim H-B, Yang M, Heo J, Won Y, Jang S S, Park J K, Son Y, Oh T I (2017). Chem Mater.

[18] Konnova S A, Lvov Y M, Fakhrullin R F (2016). Langmuir.

[19] Fakhrullin R F, Paunov V N (2009). Chem Commun.

[20] Lee J, Choi J, Park J H, Kim M-H, Hong D, Cho H, Yang S H, Choi I S (2014). Angew Chem, Int Ed.

[21] Park J H, Kim K, Lee J, Choi J Y, Hong D, Yang S H, Caruso F, Lee Y, Choi I S (2014). Angew Chem, Int Ed.

[22] García-Alonso J, Fakhrullin R F, Paunov V N, Shen Z, Hardege J D, Pamme N, Haswell S J, Greenway G M (2011). Anal Bioanal Chem.

[23] Dzamukova M R, Naumenko E A, Rozhina E V, Trifonov A A, Fakhrullin R F (2015). Nano Res.

[24] Borovička J, Metheringham W J, Madden L A, Walton C D, Stoyanov S D, Paunov V N (2013). J Am Chem Soc.

[25] Borovička J, Stoyanov S D, Paunov V N (2013). Nanoscale.

[26] Liu M, Fakhrullin R, Novikov A, Panchal A, Lvov Y (2019). Macromol Biosci.

[27] Yendluri R, Lvov Y, de Villiers M M, Vinokurov V, Naumenko E, Tarasova E, Fakhrullin R (2017). J Pharm Sci.

[28] Cavallaro G, Chiappisi L, Pasbakhsh P, Gradzielski M, Lazzara G (2018). Appl Clay Sci.

[29] Lisuzzo L, Cavallaro G, Pasbakhsh P, Milioto S, Lazzara G (2019). J Colloid Interface Sci.

[30] Konnova S A, Sharipova I R, Demina T A, Osin Y N, Yarullina D R, Ilinskaya O N, Lvov Y M, Fakhrullin R F (2013). Chem Commun.

[31] Konnova S A, Lvov Y M, Fakhrullin R F (2016). Clay Miner.

[32] Tully J, Yendluri R, Lvov Y (2016). Biomacromolecules.

[33] Lisuzzo L, Cavallaro G, Lazzara G, Milioto S, Parisi F, Stetsyshyn Y (2018). Appl Sci.

[34] Kalay S, Stetsyshyn Y, Lobaz V, Harhay K, Ohar H, Çulha M (2016). Nanotechnology.

[35] Komiyama M, Mori T, Ariga K (2018). Bull Chem Soc Jpn.

[36] Fakhrullina G I, Akhatova F S, Lvov Y M, Fakhrullin R F (2015). Environ Sci: Nano.

[37] Lee H, Hong D, Cho H, Kim J Y, Park J H, Lee S H, Kim H M, Fakhrullin R F, Choi I S (2016). Sci Rep.

[38] Burger M M (1969). Proc Natl Acad Sci U S A.

[39] Kolata G B (1975). Science.

[40] Ramanathan K, Kamalasanan M N, Malhotra B D, Pradhan D R, Chandra S (1997). J Sol-Gel Sci Technol.

